# Laser beam melting 3D printing of Ti6Al4V based porous structured dental implants: fabrication, biocompatibility analysis and photoelastic study

**DOI:** 10.1038/srep45360

**Published:** 2017-03-28

**Authors:** Fei Yang, Chen Chen, QianRong Zhou, YiMing Gong, RuiXue Li, ChiChi Li, Florian Klämpfl, Sebastian Freund, XingWen Wu, Yang Sun, Xiang Li, Michael Schmidt, Duan Ma, YouCheng Yu

**Affiliations:** 1 Department of Stomatology, Zhongshan Hospital, Fudan University, Shanghai, 200032, China; 2 Institute of Photonic Technologies, Friedrich-Alexander-Universität Erlangen-Nürnberg, Konrad-Zuse-Str. 3/5, D-91052 Erlangen, Germany; 3 Erlangen Graduate School in Advanced Optical Technologies, Paul-Gordan-Str. 6, D-91052 Erlangen, Germany; 4 Department of Plastic Surgery, The 1st Affiliated Hospital of Wenzhou Medical University, Wenzhou, 325000, China; 5 State Key Laboratory of Mechanical System and Vibration, School of Mechanical Engineering, Shanghai Jiao Tong University, Shanghai, 200240, China; 6 Key Laboratory of Metabolism and Molecular Medicine, Ministry of Education, Department of Biochemistry and Molecular Biology, Collaborative Innovation Center of Genetics and Development, Institutes of Biomedical Sciences, School of Basic Medical Sciences, Fudan University, Shanghai, 200032, China

## Abstract

Fabricating Ti alloy based dental implants with defined porous scaffold structure is a promising strategy for improving the osteoinduction of implants. In this study, we use Laser Beam Melting (LBM) 3D printing technique to fabricate porous Ti6Al4V dental implant prototypes with three controlled pore sizes (200, 350 and 500 μm). The mechanical stress distribution in the surrounding bone tissue is characterized by photoelastography and associated finite element simulation. For *in-vitro* studies, experiments on implants’ biocompatibility and osteogenic capability are conducted to evaluate the cellular response correlated to the porous structure. As the preliminary results, porous structured implants show a lower stress-shielding to the surrounding bone at the implant neck and a more densed distribution at the bottom site compared to the reference implant. From the cell proliferation tests and the immunofluorescence images, 350 and 500 μm pore sized implants demonstrate a better biocompatibility in terms of cell growth, migration and adhesion. Osteogenic genes expression of the 350 μm group is significantly increased alone with the ALP activity test. All these suggest that a pore size of 350 μm provides an optimal provides an optimal potential for improving the mechanical shielding to the surrounding bones and osteoinduction of the implant itself.

Titanium (Ti) and its alloys are considered as the preferred biomaterials for fabricating dental implants due to their high tensile strength, good biocompatibility and resultantly the presence of osseointegration^1^,^2^. However, with the development of oral implantology, concerns have been aroused for the bone resorption led by the mechanical stress shielding in bone tissue and the realization of life-time implant success rate. Furthermore, an earlier peri-implant bone formation and a better bone-implant contact should be achieved to realize the early function of the implant^3^. Thus, further research on improving the osteointegration and the mechanical stress shielding in bone tissue is essential for fabricating dental implants.

Generally accepted, porous structured Ti alloys have their utilities in osteoinduction of biomaterials^4^,^5^,^6^,^7^. They are suitable for repairing / replacing bone defections. This is mainly due to their stiffness, properties of adjustable pore size and the hierarchical architecture for bone tissue in-growth. Porous structured Ti alloys have already been used in clinical trials such as orthopedic prostheses, artificial hip joints and spinal fusion devices. A study presents that the porous structured scaffold with a 3D architecture potentially benefits cell seeding, vascularization, transport of oxygen, nutrients and metabolites^8^. It is claimed that the porous structure can provide anchorage of mineralized tissue into the pores and consequently leads to the osseo-incorporation phenomenon. This benefits the healing potential of bone growth into the implant^9^,^10^,^11^. Also, based on the *in-vitro* studies giving a critical support for the osteoinduction of porous Ti, mesenchymal stem cells differentiation towards osteoblasts is promoted with the micro-structured material^4^,^5^. Among all these studies, pore size is a decisive factor to influence the bioactivities at the porous structure during the bone tissue regeneration. Pore size ranging from 150 to 1000 μm can facilitate the bone in-growth^12^.

When dental implant surface directly interacts with bone tissue, the effective contact area of the implant surface determines the interaction between implant material and the surrounding tissue. The modification of the macro-structure of porous structured Ti (in scaffold structure) can alter its functional output on biocompatibility behavior. A scaffold structure can further enlarge the specific contact area and consequently affects the bone formation at the bone-to-implant interface. Moreover, it is concluded that a modification as a porous structure design helps to generate a lower stress-shielding to the bone tissue around the implant screw thread. Realizing this effect, numerous studies are devoted to fabricating porous Ti scaffold on dental implants and demonstrate an efficient bone in-growth into the porous structure^13^,^14^.

3D printing enables to create a complex 3D hierarchical architecture directly from a computational model. This feature promptes an alternative to fabricating porous structured dental implants. Among all types of Ti alloys used for 3D printing, Ti6Al4V is most commonly used. Since Brånemark first discovered the osseointgration phenomenon between the Ti and the bone tissue, endosseous screw-shaped dental implants made by Ti6Al4V have been mostly commercially produced and clinically used in dentistry nowadays^3^. However, providing adequate bone-implant contact and promoting the implant’s bioactivity to realize the early function and a long life-time dental implant is always under the effort of dental implant industry. So far, there is still no consensus on the optimal pore size specification for fabricating porous Ti6Al4V material^15^. Also, the achieve a high homogeneity in pore size remains challenging. Therefore, to investigate and fabricate a homogeneous pore structure in an optimal size by means of 3D printing technique would provide theoretical and experimental basis for manufacturing novel dental implants in future.

In this study, we would specifically investigate the optimal pore size for fabricating Ti6Al4V dental implants with porous scaffold structure. When fabricating porous structured Ti6Al4V dental implants, we combine the porous scaffold structure with the screw thread in a single-body by using Laser Beam Melting (LBM) 3D printing. The pre-defined external shape, as well as the internal architecture (dimension), are totally controlled. We print out 3 types of dental implants with different pore sizes (200, 350 and 500 μm) and the conventional screw-shaped implant as the control group. To distinguish our work from all other pilot studies on fabricating porous Ti dental implants, we completely investigate the functional output of the fabricated implants regarding the mechanical stress shielding effect and the material biocompatibility. Subsequently, the mechanical stress distribution in the surrounding bone is assessed by photoelastographic phantom validation. Results of the photoelastography are verified by a 2D finite element simulational description of stress distribution in implant-jaw coupling. In addition, *in-vitro* studies are conducted to evaluate the cellular response to the porous dental implant prototype. During the experiments, we attempt to observe the migration of the attached cells directly. By doing these, we can obtain an optimal pore size to benefit the mechnical stress shielding and meanwhile the biocompatibility.

## Results

### Illustration of produced implants

**Figure 1:**
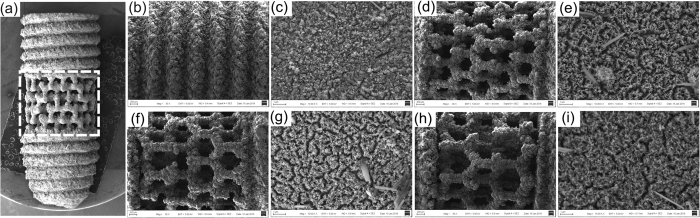
(**a**) The general pattern of one kind of porous implant. The mid-piece area of all types of implants indicated by the white frame are further observed with the SEM. The SEM micrographs of (**b**), (**c**) reference screw type implant. Porous structured implant with pore size of 200 μm in (**d**), (**e**); 350 μm in (**f**), (**g**); 500 μm in (**h**), (**i**) at low (×30) and high (×10000) magnification respectively.

Since the effect of porous structure on bone tissue formation is not fully understood and varies between studies, developing rigorously-controlled internal architectures can contribute to defining a rational design for dental implant on bone regeneration. As the result, the fabricated implants are shown in the SEM images. The generated porous scaffold structure is homogeneous regarding the pore size. Figure 1(a) shows a general pattern of one kind of porous structured implant. The mid-piece area marked with a white frame is modified into 4 types of architecture (200, 350 and 500 μm pore sized and screw-typed structure). They are first observed with the SEM at low magnification (see Fig. 1b,d,f,h). At high magnification, numerous nano-sized burr-like structures can be seen on the implant surface. This is generated during the alkali heat treatment (mentioned later in the discussion section) to achieve a further nano-modification (see Fig. 1c,e,g,i). The herein employed LBM process is effective to achieve the desired titanium implant structural modification, as the implant pores remain open after.

### Photoelastographic study

#### Comparison between reference implant and 350 μm implant

**Figure 2:**
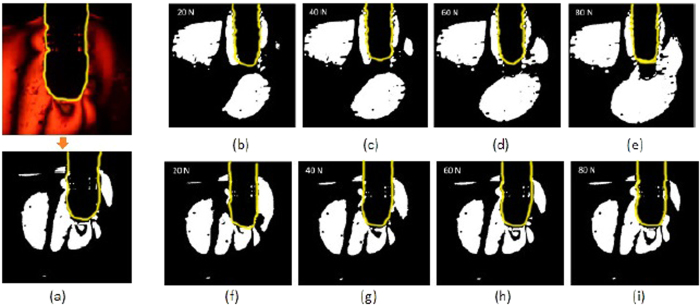
(**a**) The procedure of image post-processing converting a recorded elastograph into a binary image; Reference implant correlated to the fringe pattern under (**b**) 20, (**c**) 40, (**d**) 60, (**e**) 80 N; subfigure (**f**–**i**) parallely for fringe pattern on implant with 350 μm pore size. The manually sketched yellow line marks the layout of the implant.

As a highly concentrated distribution of mechanical stress could affect the bone-to-implant interaction, we are interested to search for high order fringes around the examined implant. Fringe pattern is presented as black-white lines in a binary image, which is converted by applying threshold on the raw image histogram (see Fig. 2a). In analyzing the effect of loading on the reference implant, a comparative analysis of variance in different normal forces is performed. As shown in Fig. 2, the fringe pattern with highest intensity is found at the bottom of the implant. Also, isochromatic pattern with retardation phase of 1 is indicated around the screw thread at the implant neck. By comparing subfigure e) to b), the 1st order fringe pattern does not propagate further. However, it is seen in subfigure e), that the 2nd fringe pattern starts to appear. The max. primary stress is 4.42 MPa (plotted from the pixel at the implant bottom site simulation model), located in the middle of the implant bottom site. Principally, the mechanical distribution is partially located around the screw thread on the reference dental implant. The placed normal force is relatively dispersed onto all positions around the dental implant.

In analyzing the effect of loading on the implant with 350 μm pore size, we find a totally different stress distribution. The fringe pattern with highest intensity is found at the bottom of the implant. When observing the fringe at the implant bottom, the oblique loading induces a significantly more densed stress distribution compared to the reference implant. It is seen in (Fig. 2i), that the 4th order fringe pattern is already generated, based on the recorded closed ring at the bottom site to represent the retardation phase 0. Compare to this, an isochromatic pattern with a lower retardation phase of 1 is indicated around the porous structure. The primary stress remains rather constant with the normal force changing from 20 to 80 N. According to the simulation results, the max. primary stress is found in the bottom site of the implant bottom and reaches 4.65 MPa. It is higher than that in the reference implant. As the brief conclusion, the mechanical stress distribution is more conducted onto the implant bottom site, compared to that on the reference implant.

#### Comparison between porous structured implants

**Figure 3:**
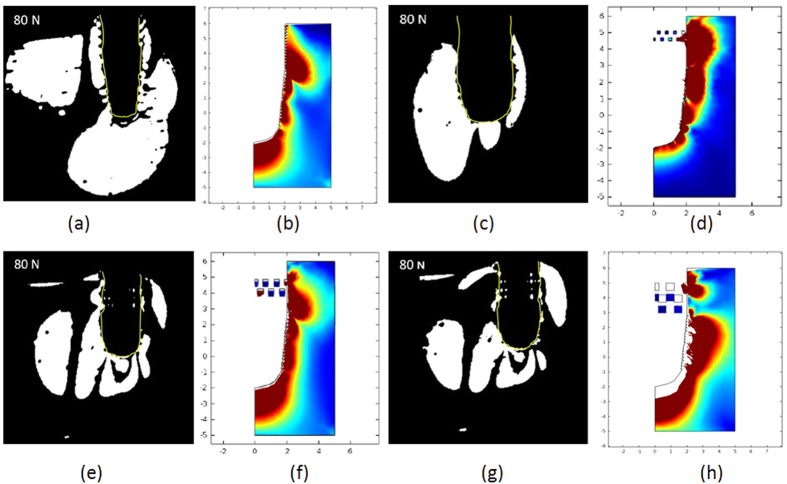
Fringe pattern under 80 N and the corresponding simulation results of (**a**), (**b**) reference implant; (**c**), (**d**) 200 μm implant; (**e**), (**f**) 350 μm implant; and (**g**), (**h**) 500 μm implant. The color calibration in the simulation results represents the relative stress from min. in blue to max. in red. The primary stress is plotted separately from the stationary studies.

In analyzing the effect of loading on implants with 200 and 500 μm pore size (experimental and simulation results shown in Fig. 3), the results are leading to similar conclusions to that on 350 μm implant. The implant with 200 μm pores shows a similar fringe pattern to the reference implant. We find fringe pattern with higher retardation phase at the implant bottom. When comparing the 500 to 350 μm implant (or 350 to 200 μm), the fringe pattern at the implant bottom is showing a higher retarding phase in its fringe order. This represents the fact that the generated primary stress at the bottom sample site is becoming higher, when the porous structure size increases. The simulation results agree to the experimental results. For the 500 μm implant, we find a max. primary stress of 6.21 MPa at the bottom sample site. For the 200 μm implant, the value decreases to 4.37 MPa.

#### Cell viability evaluation

**Figure 4:**
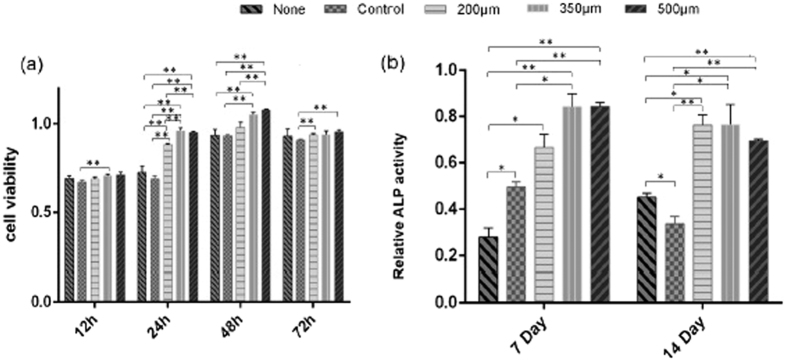
(**a**) Cell viability (**b**) and ALP activity of MC3T3-E1 cells of each group at different time points, whereby *P < 0.05; **P < 0.01.

MC3T3-E1, preosteoblast, is extracted from the mouse’s calvarial bone, which can be induced to mature osteoblast. The proliferation and differentiation of MC3T3-E1 cells on different types of implants are critical for bone tissue formation on the implants. CCK-8 assays show that MC3T3-E1 cells exhibit a significant increase in cell viability at 24 h and 48 h on 350 and 500 μm pore size structured implant, compared with all other groups at the same time period. In addition, statistical analysis shows a decrease in cell viability at 72 h due to the contact inhibition phenomenon probably (p < 0.05, see Fig. 4a).

The ALP activity of MC3T3-E1 cells on 350 and 500 μm pore size structured implant are higher than the values on the plate and that on the reference implant. The same conclusion can be drawn at day 7 and day 14. Furthermore, at day 14, the ALP activity of MC3T3-E1 cells of 200 μm group is increased compared to that on the reference implant (p < 0.05, see Fig. 4b). The porous structured implants enhance ALP levels to over 2 times higher than that of the reference implant. It is noticed that MC3T3-E1 cells on 350 and 500 μm pore always maintain a better osteogenic activity until day 14, compared with the reference implant.

#### Cell observation and migration

**Figure 5:**
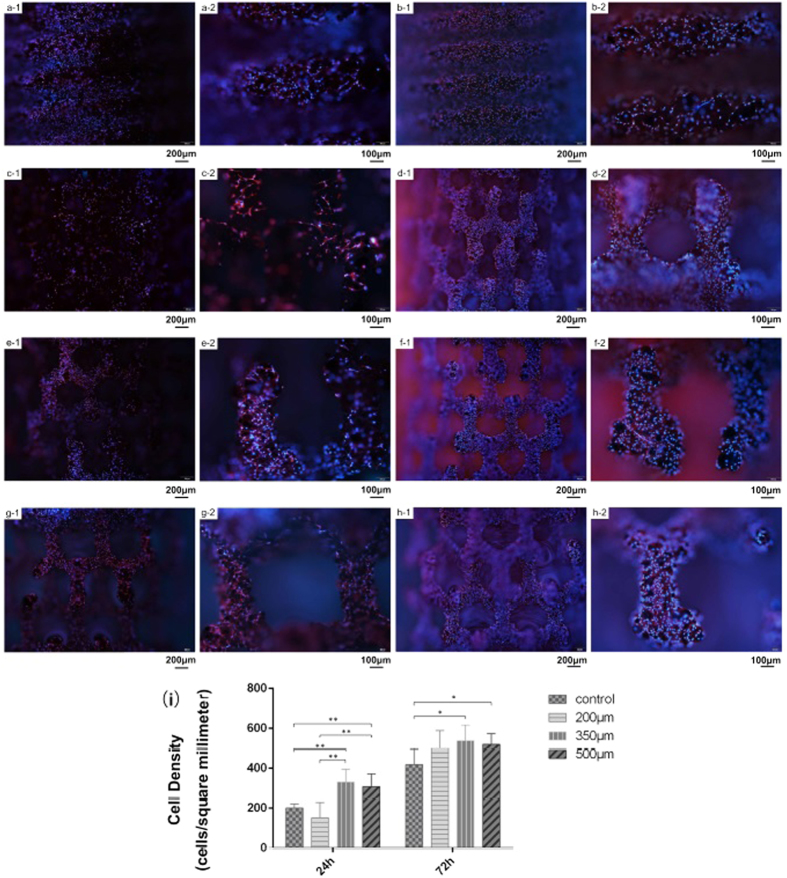
Attached MC3T3-E1 cells showed by immunofluorescence after co-cultured with different type of implant for 24 h and 72 h. Typical images of control group (a-1, a-2) for 24 h and 72 h (b-1, b-2) at low (×40) and high (×100) magnification; 200 μm group (c-1, c-2) for 24 h and 72 h (d-1, d-2) at low (×40) and high (×100) magnification; 350 μm group (e-1, e-2) for 24 h and 72 h (f-1, f-2) at low (×40) and high (×100) magnification; 500 μm group (g-1, g-2) for 24 h and 72 h (h-1, h-2) at low (×40) and high (×100) magnification. i) Cell density of MC3T3-E1 cells attached on different type of implant at each time points. *P < 0.05; **P < 0.01.

The implant is impermeable to the light and is presented as a dark background under the optical microscope. The immunofluorescence makes MC3T3-E1 cells visible, when they attach to the implant surface. Figure 5 show the attached cells at 24 h (a-1, c-1, e-1, g-1: at low magnification; a-2, c-2, e-2, g-2: at high magnification) and 72 h (b-1, d-1, f-1, h-1, at low magnification; b-2, d-2, f-2, h-2, at high magnification). The cell density (see Fig. 5i) increases with culture time for all groups of implants. The 35 and 500 μm pore size structured implants exhibit significant rise at 24 h, compared with all other groups.

The quantitative results of cell density are verifying the cell viability test indicated hereinabove. MC3T3-E1 cells on 350 and 500 μm pore size structured implants show a high density of 331.6 and 308.5 cells/*mm*^2^ respectively at 24 h. This is more than that of the reference implant and 200 μm group. At 72 h, the 350 and 500 μm pore size structured implants (538.1 and 512.4 cells/*mm*^2^ respectively) statistically show a higher cell density than the control group (418.5 cells/*mm*^2^). Meanwhile, the cell density of the 200 μm group increases to 499.6 cells/*mm*^2^, which approaches to the other two porous structured group. In general, a higher density of MC3T3-E1 cells are attached on the porous structured implants, compared with the screw-typed implant along with the culturing time.

**Figure 6:**
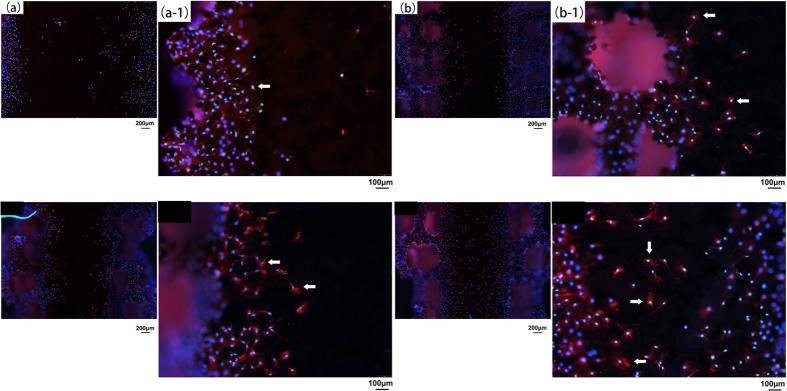
Attached MC3T3-E1 cells at the back side of different type of implant on Day 7. Few cells has migrated to the central axis of the control (a, a-1 for high magnification); 200 μm (b, b-1 for high magnification); 350 μm (c, c-1 for high magnification) group of implant. Plenty of MC3T3-E1 cells can be observed at the central axis of 500 μm pore sized implant (d, d-1 for high magnification). White arrows show the typical morphology of the attached MC3T3-E1 cells on each type of implant.

Since the implant is semiterete, the back side of the implant does not contact with MC3T3-E1 cells at the beginning. After cells anchore to the implant surface, they begin to migrate to the back side. From Fig. 6, there are few cells observed at the central axis of the control (a, a-1), 200 μm (b, b-1) and 350 μm (c, c-1) group of implant on day 7, while more amount of cells are found at the central axis of the 500 μm pore size structured implant (d, d-1). Furthermore, the back side of all types of the porous architecture are covered with dense MC3T3-E1 cells. At a high magnification, more spindle and fibrous shaped cells (indicated by the white arrows) are attached to the porous structured implant, whereas the porous structure gives the cells a more stretched shape than the reference implant according to our understanding.

#### Genic expression

**Figure 7:**
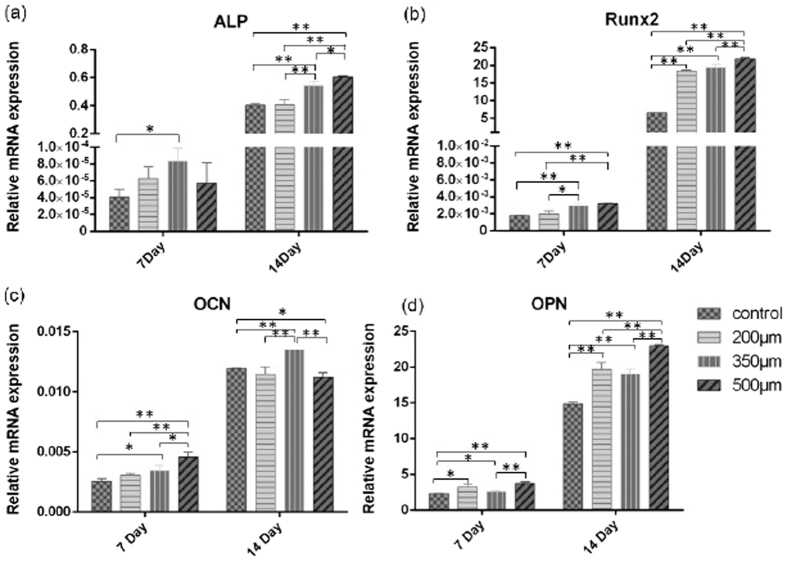
Relative mRNA expression of ALP in (**a**); Runx2 in (**b**); OCN in (**c**); and OPN in (**d**), whereby *P < 0.05; **P < 0.01.

Apart from osteoblast proliferation, migration and adhesion, cell differentiation also plays an important role in bone generation. As shown in Fig. 7, the gene expressions of ALP, Runx2, OCN and OPN are evaluated by qRT-PCR. In general, all associated values increase with culture time. After 14 days, all the gene expressions in both 350 and 500 μm groups are higher than that in the control group (see Fig. 7). The gene expressions of ALP, Runx2, and OPN in the 500 μm group are in a significantly upper level to the other groups’ (see Fig. 7a,b,d). On day 7, the gene expression of Runx2 in both 350 and 500 μm groups rise faster than the other two groups. On day 14, the Runx2 expression in all of the porous structured implants are becoming higher than that in the control group (see Fig. 7). As a later marker of osteoblastic differentiation, OCN is highly expressed in the 350 μm group on day 14 compared to the others (see Fig. 7c). As a regulative molecule of cell adhesion, OPN increases in all porous structured implants (200, 350 and 500 μm group), compared with that of the reference implant, at both day 7 and day 14 (see Fig. 7). This is consistent with the cell immunofluorescence observation hereinbefore.

## Discussion

### Selection of fabrication procedure and specifications

Selecting a proper fabrication procedure for producing porous scaffold structure on a dental implant is difficult. It is mainly because generating homogeneous pore size in Ti6Al4V material is challenging. There are various procedures that we could have chosen to fabricate porous structure, such as microspheres sintering^5^, slurry forming method^4^ etc. Unfortunately, all these processes exhibit a common disadvantage, that the porous structure is inconsistent in pore size and resultantly the biocompatibility evaluation at the surface of the scaffold is limited. As the most recent fabrication procedure, the LBM 3D printing technique seems to be suitable to fabricate a dental implant with homogeneous pore size. Also, compared to the conventional fabrication (such as CNC maching), 3D printing provides a more cost-efficient solution to produce patient-specific implant design to compromise bone volume and tailor the interior porous architecture. By fabricating porous Ti6Al4V dental implant prototype with variant pore sizes, we demonstrate an alternative for producing customized dental implant in the future.

Similar to our work, Li *et al*. presented the integration of computational design and additive manufacturing technology. They also successfully applied the 3D printing to fabricating a porous Ti6Al4V scaffold structured^16^. Also, conclusions of other established researches give a positive support for fabricating porous structure on Ti6Al4V implant. However, selecting the optimal pore size remains an open question and there is no direct answer correlated to the specific need on porous scaffold structured dental implant. It is only proved that osteoblasts’ adhesion and proliferation are promoted if the pore size is larger than 150 μm. Based on the conclusions, we strictly control the pore size during computational design and present three types of porous implants with 200, 350 and 500 μm pore size. Combined with the succeeded generation of homogeneous pore size, we can further investigate the mechanical stress shielding on the surrounding bone tissue and the biocompatibility of implants with different pore sizes.

### Necessity for surface heat treatment

Surface modification is an essential process that modifies the surface function of biomaterials through changes in their chemical composition, micro-structure and morphology. At the same time, the bulk mechanical properties of the material are not affected^17^. It has been recently shown that soaking the Ti alloys in NaOH solution followed by heat treatment can accelerate the hydroxyapatite formation onto the materials’ surface and bone-like apatite. This could be formed, when they immerse in simulating body fluid (SBF) via formation of titana hydrogel on the surface. With this simple thermo-chemical heat treatment, the complex porous structure achieves a surface uniform modification in contrast with other surface processing techniques, including ion implantation, electrochemical treatments and hydrothermal treatments^18^. Also, for the purpose of parallely comparing the biocompatibility in porous structured implant to the screw-shaped reference implant, we employed the alkali heat treatment on all implants. As the result, we obtain a consistent surface modification at both of the screw-shaped area and the porous architectures. Taken together, we suggest that the LBM 3D printing and alkali heat treatment are practicable for fabricating a complicated, highly precise structured Ti6Al4V implant.

### Stress distribution in surrounding bone

The stress-shielding around the edentulous region of dental implant can usually lead to some complications such as bone resorption or loss of osseointegration. In fact, it is generally reported that high peak stresses around the neck of dental implant can increase the marginal bone loss after operation and even loosen the bone-implant interface^19^,^20^,^21^. Therefore, better knowledge of the porous implant’s mechanical distribution is needed for evaluating the feasibility of the porous dental implant in clinical use. Most importantly, how the porous architecture loads its stress to the surrounding bone after the bone tissue in-growth is the main concern after the morphological alteration of the dental implant.

As the fringe pattern of the photoelastography suggests, the mechanical stress distribution is partially located around the screw thread of the reference dental implant. In contrast, less primary stress can be off-set by the host material in scaffold structure on porous structured implants with its size increasing from 200 to 500 μm. The generated primary stress under 20, 30, 40 and 80 N are showing the same tendency (see Table 1). Notice here, the plotted values for stress at implant neck are estimation only because of the variance in the biomechanical specifications cortical bones between different researches. Also, the plotting points on models with different porous structures are slightly different. When observing the fringe pattern at the bottom site of porous structure implant, considering the distance of this fringe line to the implant, we would suppose that fringe line with higher order (such as 4 -5) is spread onto the constrained side of the phantom. Principally, the mechanical force distribution is not localized around the scaffold structure of the implants.

Lai *et al*. claim in their study on stress distribution around a single osseointegrated implant that high stresses in bone are always located around the neck of the conventional implant^22^. Another study compares load on solitary implants with load on 4 implants connected with a bar in the interforaminal region of the mandible. It is found that the most max. primary stresses are located around the neck of the implants. Here in our experiments, we would only partly agree to these conclusions. As we verify in the reference test, that normal force is contributed to both the screw thread on the neck of the reference implant, as well as to the bottom sample site. In the case of the porous structured implants, authors observes a reduction in the magnitude of the principal stresses that occurred around the neck of the implants, compared to the reference implants. In fact, primary stresses are mostly conducted to the bottom sample site of the porous structured implants.

### Biocompatibility improvement

#### Evaluation of cell proliferation

From the cell viability test, it is noticed that MC3T3-E1 cells present a higher proliferation rate on porous structured implant, compared to reference implant. Also, the proliferation rate is apparently higher than the rate on the plate without any implant at the rapid growth period, which is consistent with St-Pierre’s study in porous Ti scaffolds^23^. However, the cell proliferation assay does not explain whether there is any MC3T3-E1 cell located at the inner pore regions, while the location of osteoblasts is essential for bone regeneration. To ensure the cells’ location, immunofluorescence is employed in our study and makes the cell proliferation observation better visualized. The result of cell density is mainly consistent with the cell viability test that MC3T3-E1 cells on porous structured implant present a higher proliferation rate. Besides, we notice that MC3T3-E1 cells in the 200 μm group exhibit an inferiority in cell density compared to those in the 350 and 500 μm groups initially and tend to be approaching after.

The transport of oxygen and nutrients is requisite for cell proliferation^9^. The specific contact area of the porous implants is enlarged till the appropriate pore size induces the oxygen and nutrients’ transport inside the porous regions. As for the 200 μm pore sized implant, a less-opened pore structure is generated compared with the 350 and 500 μm pore sized implants. This could lead to an insufficient soaking with the culture medium at the inner pore region because of the capillary effect on the pore opening. As the output on biocompatibility, a lower cell density is presented at the first 24 h. After the permeation of the culture medium, MC3T3-E1 cells at the inner pore region begin to grow rapidly. Furthermore, as the specific surface area of 200 μm pore sized implant is larger than the 350 and 500 μm implant, the cell density of all the porous implant group seems to approach to each other at the later 72 h. The presence of the porous structure is expected to enlarge the specific contact area for the transport of oxygen and nutrients. This explains the consequence of induced cell proliferation on the porous implants. In this study, we intend to have a deep insight of the cell proliferation promoting causes, since the alteration of the implants’ geometric structure is executed. We pre-sume that the porous structure gives the MC3T3-E1 cells an interconnected architecture to improve the cell migration.

#### Evaluation of cell migration ability

In the previous work, Matena J use the migration factors to evaluate the osteoblasts’ migration potential, because it was difficult to observe the direct migration despite the cells seeding in the porous structures^24^. We would illustrate the direct cell migration ability. As for the experimental strategy, we design a porous structured semiterete implant for *in-vitro* study. As mentioned, the back side of the implant does not contact with MC3T3-E1 cells at the beginning. Only after cells anchore to the implant surface, they begin to migrate to the back side. The existence of any cells at the central axis of the implant would indicate the migration ability of MC3T3-E1 cells on different types of implant. Moreover, MC3T3-E1 cells immunofluorescence marked by vinculin protein present a stronger staining and more stretched shape on the porous implant. The degree of vinculin staining on the porous structured implant, as a critical protein for cell attachment, migration and actin cytoskeleton formation^25^, is greater than the reference implant. We speculate that a single alteration of the porous structure can still have a biological influence on Ti material by improving cell adhesion and migration. In view of all the immunofluorescence results, 350 and 500 μm implants show a better improvement in cell proliferation and migration.

#### Influence of porous structure on cell differentiation

Osteoblasts are osteogenic cells that are associated with bone formation through their production of osteoids and subsequent mineralization of the osteoid matrix. Although all the implants’ surface is modified by the alkali heat treatment, we would still investigate the influence of porous architecture on cell differentiation. Genic expression of ALP is a marker of cell differentiation in the initial stages of osteopoiesis to release inorganic phosphate to promote mineralization of the extracellular matrix^26^. It increased along with culturing time but contrary to the ALP activity test result. In osteoblast, the generation of inorganic phosphate for mineralization can also be formed by phosphate esters, such as *β*-glycerophosphate^27^. Several studies observed the similar outcomes as the inhibition of ALP activity, under the condition of up-regulation in genic expression^28^,^29^. According to these studies, there is more mineral matrix (not only inorganic phosphate) deposited in this porous topography before osteoblast differentiation.

RUNX2 is a central mediator that executes signals from the BMP and Wnt pathways to promote cell phenotype commitment and osteogenesis^30^. OCN is a later marker of osteoblastic differentiation. Alone with ALP, all these three genes’ expression shows a greater degree in 350 and 500 μm than the control group at the same time period. This may contribute to improving nutrients transport caused by the porous architecture with the appropriate pore size. As a regulative molecule of cell adhesion, OPN is showed significantly increased in the porous implant group compared with the control group, which is consistent with the cell immunofluorescence observation hereinbefore.

## Outlook

In this work, we have established our porous structured Ti6Al4V based implants. We have confirmed its perspective in clincal application by terms of photoelastic studies and biocompatibility analysis. We would further our research on the following directions. Firstly, we would implement metallorgraphic studies for more completed material characterization. We would study the cross-sectional metallography of fabricated implants to investigate the defects (closed pores and thermal crack) induced during the LBM 3D printing procedure. Secondly, a 3D contact simulation model will be constructed to achieve a better understanding of mechanical distribution in the surrounding bone tissue. This will help to improve the geometry of the implant design to better prevent the mechanical shielding. Lastly, a further research in adhesive mechanism of porous structured implant would help to promote the biocompatibility of dental implant. The design of this dental implant is sofar regarded as an applicable prototype. More efforts will be taken to define an eligible clinical used dental implant including the internal thread structure fabricating and *in-vivo* studies.

## Conclusion

In this work, we establish Ti6Al4V based dental implants with porous scaffold structure. The generated porous structure is homogeneous in size. In terms of the fabrication procedure, LBM 3D printing and alkali heat treatment are practicable for fabricating a complex and highly precise structured dental Ti6Al4V implants. The biocompatibility of the implants is likely improved by porous scaffold structure compared with conventional screw-type implant. MC3T3-E1 cells on the 500 μm pore size structured implant shows the best capacity of migration and both 350 and 500 μm pore sized implant present a notable improvement in cell proliferation, attachment and differentiation. In consideration of the photoelastic studies and *in-vitro* analysis, the 350 μm pore sized implant has the best mechanical stress distribution in the surrounding bone tissue and a satisfied biocompatibility. All these conclusions are suggesting a future perspective of using the porous structured Ti6Al4V dental implants in clinical trails within further improvement on implant geometry and implementation of animal/human tests.

## Methods

### Laser Beam Melting 3D printing of dental implant

#### Implants fabrication

**Figure 8:**
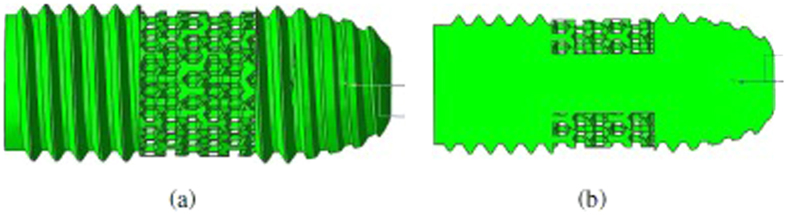
The intended design of porous structured implant of (**a**) front and (**b**) back side in cross-sectional view

To investigate the effect of porous structured implants *in-vitro*, semiterete implant was designed to 1 reference screw structure and 3 types of porous structure (pore size of 200, 350 and 500 μm) in the mid-piece of the implant with 1 mm thickness. The half implant was 10 mm in length and 4.5 mm in width. Considering the taping effect of the implant bottom and the space for the inner screw to link up with the abutment for further clinical application, we placed the porous structure at the mid-area of the implant with 3 mm in length (see Fig. 8). In this study, we proposed a modern technique, LBM 3D printing, for controlling the pore size during the fabrication of porous Ti6Al4V implant. Screw thread and porous structure of implants were pre-defined by NX Unigraphics and layered printed with the selective laser melting solution. After LBM fabrication, Ti6Al4V could reach an elongation at break of 5–7%. For LBM solution, we used an SLM 50 LBM machine (Trumpf GmbH, Germany). It features a rotational recoater with a flexible lip (TLS technik GmbH, Germany) in contact with the powder and a single-mode Yb:YAG fiber laser with a wavelength of 1075 nm, a laser kerf of 50 μm and a maximum output power of 400 W. The process chamber was flushed with argon gas to prevent the material from oxidation. Laser kerf was minimized to 10 μm by hatching the working distance of laser beam.

#### Surface processing and characterization

The prepared dental implants with different morphologies was finished by the alkaline heat treatment in NaOH solution [5 mol/l]. The samples were treated at 600 °C for 1 h. This alkaline heat treatment procedure is done for the purpose of bioactiviation modification. All implants were cleaned by ultrasonic wave for 20 min in distilled water, acetone solution and 70% ethanol and autoclaved at 120 °C for 40 min after the heat treatment. After the final heat treatment, the morphology of implant surface and the porous structure were observed by the Field Emission Scanning Electron Microscopy (FE-SEM, ZEISS, Germany).

### Photoelastographic study

#### Photoelasticity analysis

We built a monochromatic polariscope as the experimental setup for photoelastography. The experiment was performed in 2D because this method is more commonly used and more friendly to computational power during the imaging post-processing. It consisted of 2 linear polarizers, 2 quarter waveplates (Thorlabs, Germany), an LED light source (*λ* = 630 nm) and 1 CCD camera. Normal force was placed perpendicularly onto the implant to mimic the biting force. The forces ranged from 20, 30, 40 over 50, 60, 70 to 80 N. The photographic records obtained from each load application were stored in an image database. A visualization of the intensity of the stresses (fringe order: 0, 1/2, 1, 3/2, 2, 5/2, etc. in the different regions of each model was performed. We validated the mechanical force distribution around the implant through the 2D photoelastic experiments on bone mimicking tissue phantom. The phantom was constructed with castable aliphatic polyurethanes (BJB Enterprise Co., US). The finished PU material has an average density of 1050 kg/*m*^3^ and a Young’s moduli of 820.42 MPa (determined by DIN-ISO 527 universal mechanical test). The PU liquid mixture were casted into a thin mold, where the half implant was folded. This simulates the geometry, where the dental implant is coupled into the jaw.

#### Finite element simulation

To check the experimental results, as well as to qualitatively determine the magnitude of stress, a 2D finite element model was constructed by using COMSOL (V5.2a, COMSOL Multiphysics; CPU:Intel(R) Core(TM) i7-6700HQ) based on the physical properties of the implant and the surrounding bone. The cross sectional model included the shapes as follows: 1. Reference implant 2, Friadent 500 μm 3. Friadent 350 μm, 4. Friadent 200 μm under the normal force as implemented during the photoelastographic experiments. The geometry of the implant model was simplified from the experimental implants. Domains for bones demonstrated a solid pattern within the experimental model of the PU phantom. A fixed bond (complete load transfer) between the bone and the implant was set over the entire interface. Direct contact between the friadent and the bone tissue was assumed. The complete contact was simulated by alligning contact pair and fixed constrain. The specification for the Young’s moduli in the defined contact physics was obtained by the mechanical test on the PU phantom matrix. The geometry of the reconstructed physics was meshed by triangular nets before the stationary study.

### Biocompatibility studies

#### Cell culture

The osteoblast-like cell line MC3T3-E1, Subclone 14, was obtained from the cooperating institution(Shanghai Cellular Institute of China Scientific Academy) and cultured in *α*-MEM medium (Minimum Essential Medium *α*,Gibco, US) supplemented with 10% fetal bovine serum (FBS, Gibco, US) and 1% Penicillin/Streptomycin (Penicillin, Gibco, US) at 37 °C in a 5% *CO*_2_ incubator.

#### Cell viability test

To assess the proliferation rate of MC3T3 cells when they were either or not co-cultured with the implants, a cell viability test was conducted. The cell viability was evaluated by a cell counting kit-8 (CCK-8) assay (Vazyme Biotech, Nanjing, China) according to the manufacturer protocol. MC3T3-E1 cells were seeded on the plate (None) or the implant surfaces in 24-well plates at a density of 2 × 10^5^ cells ml-1 and cultured for 12, 24, 48 and 72 h and at the each time points, the cells were incubated with 600 *μ*L *α*-MEM and 60 *μ*L CCK-8 solution in each well for 2 h at 37 °C. The optical density (OD) was immediately measured at the wavelength of 450 nm using a microplate ELISAs reader (SUNRISE, Tecan, Switzerland).

#### Alkaline phosphatase (ALP) activity

In order to investigate the differentiation of the osteoblasts, the alkaline phosphatase (ALP) activity of the cells on different types of implants was evaluated by an ALP assay kit (Beyotime, Nanjing, China) according to the manufacturer’s protocol. MC3T3-E1 cells were seeded on the plate (None) or the implant (control 200, 350 and 500 μm) surfaces in 24-well plates at a density of 1 × 10^5^ cells ml-1 and cultured for 7 and 14 Days.

#### Cell morphologies

Although the implant is impermeable to the light, a visualized observation is still needed for a more intuitional purpose. During the experiment, MC3T3-E1 cells were seeded on implant surfaces in 24-well plates at a density of 1 × 10^5^ cells ml-1 and cultured for 24 hours and 3 Days. After removing the suspended cells with PBS, the attached cells were fixed with acetone and methyl alcohol solution and blocked with 5% FBS for 1 h. Implant was incubated with primary antibody against vinculin(1:100, Abcam, Cambridge, US) at 4 overnight and then incubated with Cy3-conjugated goat antirabbit secondary antibody(1:1000) at room temperature for 1 h. Lastly, nuclei were counterstained with 4′,6 diamidino-2-phenylindole (DAPI). The immunofluorescence were observed with an optical microscope (BX53, OLYMPUS, Japan).

#### Quantitative real time PCR (RT-PCR)

To evaluate the differentiation and maturation of MC3T3-E1 cells is critical for indicating the bone tissue formation on the implant. To do this, a qRT-PCR test was conducted. MC3T3-E1 cells were seeded on implant surfaces in 24-well plates at a density of 1 × 10^5^ cells ml-1 and cultured for 7 and 14 Days. Total RNA was extracted from the attached cells (5 implants in 1 group) by using TRIzol reagent (Invitrogen Life Technologies, US) and converted into cDNA subsequently by using PrimeScript RT master Mix (Takara, Dalian, China). A SYBER Premix Taq II kit (Takara, Dalian, China) was used for RT-PCR, which was performed in a 25 *μ*L reaction volume using the ABI PRISM 7500 sequence detection system (Appiled Biosystems, California, US) following the recommended protocol for SYBR green. Relative transcript levels were measured and normalized with GAPDH levels.

The gene expression levels of alkaline phosphatase (ALP), runt-related gene 2 (Runx2), osteocalcin (OCN), osteopontin (OPN) and GAPDH were detected using the following primers: forward primer 5′-ctgactgacccttcgctctc-3′ and reverse primer 5′-tcatgatgtccgtggtcaat-3′ for ALP; forward primer 5′-cgacagtcccaacttcctgt-3′ and reverse primer 5′-cggtaaccacagtcccatct-3′ for Runx2; forward primer 5′-ttctgctcactctgctgacc-3′ and reverse primer 5′-accttattgccctcctgctt-3′ for OCN; forward primer 5′-tctgatgagaccgtcactgc-3′ and reverse primer 5′-aggtcctcatctgtggcatc-3′ for OPN and forward primer 5′-tgctggtgctgagtatgtggt-3′ and reverse primer 5′-agtcttctgggtggcagtgat-3′ for GAPDH. Relative expression of a specific gene was calculated by using the comparative Ct method.

### Statistical analysis

All experiments were repeated at least three times and the experimental data were presented as the mean value ± SD. Student’s t-test was performed to compare among raw result groups. In all cases, differences with p < 0.05 were considered statistically significant.

## Additional Information

**How to cite this article:** Yang, F. *et al*. Laser beam melting 3D printing of Ti6Al4V based porous structured dental implants: fabrication, biocompatibility analysis and photoelastic study. *Sci. Rep.* **7**, 45360; doi: 10.1038/srep45360 (2017).

**Publisher's note:** Springer Nature remains neutral with regard to jurisdictional claims in published maps and institutional affiliations.

## Figures and Tables

**Table 1:** The max. primary stres at the neck and the bottom site of variant implant under the load of 20, 30, 40 and 80 N.

	Reference implant	200 μm pore implant	350 μm pore implant	500 μm pore implant
Bottom under 80 N	4.42 MPa	4.37 MPa	4.65 MPa	6.21 MPa
Bottom under 40 N	2.67 MPa	2.59 MPa	2.97 MPa	3.78 MPa
Bottom under 30 N	2.03 MPa	1.98 MPa	2.58 MPa	3.26 MPa
Bottom under 20 N	1.35 MPa	1.32 MPa	1.79 MPa	2.46 MPa
Neck under 80 N	1.73 MPa	1.44 MPa	1.08 MPa	0.87 MPa
Neck under 40 N	1.19 MPa	0.85 MPa	0.57 MPa	0.48 MPa
Neck under 30 N	0.75 MPa	0.71 MPa	0.43 MPa	0.36 MPa
Neck under 20 N	0.64 MPa	0.52 MPa	0.39 MPa	0.27 MPa
